# Sudden Infant Death Syndrome: Risk Factors and Newer Risk Reduction Strategies

**DOI:** 10.7759/cureus.40572

**Published:** 2023-06-17

**Authors:** Anita Vincent, Ngan Thy Chu, Aashka Shah, Chaithanya Avanthika, Sharan Jhaveri, Kunika Singh, Om M Limaye, Himasaila Boddu

**Affiliations:** 1 Medicine and Surgery, Karnataka Institute of Medical Sciences, Hubli, IND; 2 Paediatrics, City Children's Hospital, Ho Chi Minh city, VNM; 3 Paediatrics and Child Health, Pramukhswami Medical College, Karamsad, Anand, IND; 4 Pediatrics, Icahn School of Medicine at Mount Sinai, Queens Hospital Center, New York City, USA; 5 Medicine and Surgery, Smt. Nathiba Hargovandas Lakhmichand Municipal Medical College (NHLMMC), Ahmedabad, IND; 6 Paediatrics, Xinjiang Medical University, Xinjiang, CHN; 7 Paediatrics, Lokmanya Tilak Municipal Medical College and Sion Hospital, Mumbai, IND; 8 Paediatrics, Dr. Pinnamaneni Siddartha Institute of Medical Sciences and Research Foundation, Krishna, IND

**Keywords:** and neonatology, pediatrics, suid, sids, sudden infant death

## Abstract

Sudden infant death syndrome (SIDS) continues to be one of the top causes of infant death in the U.S. Despite significant public health initiatives focused on high-risk populations to enhance sleep environments and techniques. The SIDS rate has remained stable in recent years. Risk factors and newer risk reduction strategies for SIDS are the focus of this review article. We conducted a comprehensive literature search on Medline, Cochrane, Embase, and Google Scholar until July 2022. The following search strings and Medical Subject Heading (MeSH) terms were used: "SIDS," "Sudden Infant Death" and "SUID". We explored the literature on SIDS for its epidemiology, pathophysiology, the role of various etiologies and their influence, associated complications leading to SIDS, and preventive and treatment modalities. Despite a more than 50% drop-in rates since the start of the "Back to Sleep" campaign in 1994, sudden infant death syndrome (SIDS) continues to be the top cause of post-neonatal mortality in the United States, despite continued educational initiatives that support safe sleep and other risk reduction strategies. The new American Academy of Pediatrics guidelines for lowering the risk of SIDS include a lot of emphasis on sleeping habits, bedding, and environment but also include elements that are frequently ignored (i.e., prenatal care, smoking, alcohol and drug use, and childhood vaccinations). This study highlights these less-frequently discussed aspects and identifies treatments that have produced beneficial behavioral shifts that benefit newborns as well as their mothers' health and wellbeing.

## Introduction and background

Sudden Infant Death Syndrome (SIDS) accounts for about 38.4 deaths per 100,000 live births (approximately 1,389 deaths) as per records of the year 2020 [1]. In the majority of the countries, there was a rapid surge in the cases of SIDS in the early 1980s followed by a decline in the 1990s. The main reason for this dramatic deterioration in the cases was the "Back to Sleep" campaign which was set in motion by the American Academy of Paediatrics in 1994, which raised awareness regarding a healthy sleeping environment [2]. Despite this, the past few years have seen a dramatic plateau in the rate of SIDS and it is still a notable cause of death for infants under the age of one year [3]. All these statistics suggest a multifactorial association in the causation of SIDS.

SIDS can be defined as "The sudden death of an infant under one year of age which remains unexplained after a thorough case investigation, including the performance of a complete autopsy, examination of the death scene, and review of the clinical history" [4]. A large number of cases of SIDS are seen during the second, third, and fourth months of life (~90%) and are more prevalent amongst males as compared to females (3:2) [5]. SIDS clinically presents as an unanticipated death of an infant which occurs during sleep, particularly if the child is sleeping in a decumbent position. SIDS falls under a much larger category known as Sudden Unexpected Infant Death (SUID) which is defined as 'a sudden and unexpected death, whether explained or unexplained, occurring during infancy' [6]. 

When the cause of death remains unidentified and cannot be pointed toward any of the known causes of death (strangulation, suffocation, medical condition, or asphyxia) it is considered a case of SIDS [6]. Apart from sleeping practices like sleeping in a recumbent position, bed sharing with adults, and avoidance of room sharing, which has proven to be a major game-changer in the incidence of SIDS, several other risk factors have also been identified to be associated with SIDS. These include extrinsic factors like exposure to cigarette smoke, alcohol and drug consumption, bottle feeding, and intrinsic factors like demography, gender, premature birth, and intrauterine pathology [7-9].

It is important to understand how crucial each of these risk factors is in causing SIDS, and therefore the main objective of this review is to identify the majority of risk elements associated with SIDS and outline various modes of prevention and intervention in the reduction of infant mortality rate due to the same.

## Review

Pathophysiology

**Figure 1:**
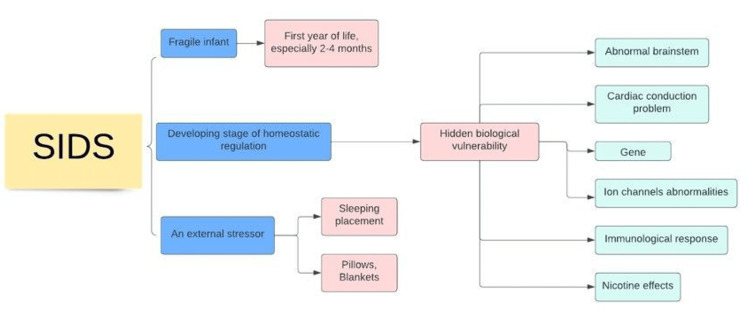
SIDS pathophysiology SIDS: Sudden infant death syndrome

SIDS is considered a condition with unknown reasons even after autopsy because the pathogenesis of SIDS is combined by many conditions, including genes, environment, and socio-culture. Figure 1 describes the pathophysiology of SIDS. Three factors, including a fragile infant, a developing stage of homeostatic regulation and an external stressor(s) are suggested to play a role in SIDS [10]. 

This hypothesis concluded that an infant must first have an underlying condition and then be influenced by an external source, such as an inappropriate sleeping position. Finally, to cause SIDS, all three need to happen together, meaning the stress must occur in a fragile infant in the first year of life, especially in the second to a fourth-month-old. The last components in the model have been well researched and used to educate the parents widely, but the vulnerable condition remains to be identified.

A vulnerable infant could be one with hidden biological vulnerability, such as the abnormal brainstem, cardiac conduction problems, gene, ion channel abnormalities, immunological response, and the nicotine effect on the immature brain [11]. We have discussed the genetic pattern in a separate part. Understanding brainstem abnormality would explain why SIDS occurs when a baby is sleeping. The age when SIDS happens the most is the infant age when most of their sleep is in the rapid eye movement (REM) stage. Different mechanosensory airway and chemosensory autonomic responses, which are essential for survival, are dysregulated during this stage of sleep [12,13]. 

Since the afferent inputs to mechanosensory pathways drive the genioglossus (an extrinsic muscle in the tongue) to physically activate to keep the upper airways open during inspiration, the dysregulation of these pathways would be harmful to babies [14-17]. The role of airway disturbance and collapse during sleep is considered one of the mechanisms contributing to SIDS [18,19]. Moreover, during REM sleep, a decrease in the activity of neurons secreting serotonin (5-HT) or norepinephrine [20] makes disturbances in serotonergic and noradrenergic mechanisms occur, and it is blamed as an important factor that makes a child vulnerable to SIDS [21-25]. These are the reasons why SIDS would not happen when a child is awake or in an older child when his motor behaviours are well developed.

An immune reaction is a crucial additional cause of SIDS [26]. Different studies are showing how SIDS could make the mucosal immune system stimulated [27,28]. There are some SIDS babies with increased density of immunoglobulin (Ig)G and IgA immune cells in the tonsils [28]. Additionally, salivary glands have been found to exhibit increased expression of HLA class I and II as well as higher expression of CD45+ stromal leukocytes and variable expression of human leukocyte antigen-antigen D (HLA-DR) [29].

SIDS has been linked to additional factors, including bacteriological findings of *Staphylococcus aureus* infections and staphylococcal endotoxins, and smoking [30]. Nicotine has been identified recently as a factor that could metabolise genes and become a SIDS cause [10]. This finding emphasises the significance of smoking cessation counselling in SIDS prevention efforts.

The interaction of internal (as mentioned above) and external elements (sleeping placements, pillows, blankets) could lead to a life-threatening situation during sleeping time. There are protective mechanisms during these episodes as if they fail to do their jobs, it would cause unexpected death. On the other hand, if one of these characteristics is not present, SIDS is less likely to happen [31,32].

Risk factors

Demographic Factors

Sudden infant death syndrome is uncommon until 2 to 4 months when it is the highest, following which it declines. Around 90% of deaths happen within six months of age. At a 60:40 ratio, males are more inclined to die than females [33]. In the last 20 years, SIDS incidence has dropped by more than 50%, partly due to the "Back to Sleep" campaign [34]. Relative to the 3500 newborns that die every year in the U.S. from sleep-related reasons, including sudden infant death syndrome, a black baby dies in Indiana every 13 hours (SIDS) [6]. In 2013, the infant mortality rate for all infants was 7.2 deaths per 1000 live births, whereas it was 15.3 for Black infants [35]. Male population groups, including non-Hispanic black infants, American Indian or Alaska Native infants, and preterm are also intrinsic risk factors [36,37]. Although the most common sleeping position is supine among Native American, Alaska Native, Aboriginal Australian, and New Zealand Maori infants, these infants being exposed to increased levels of smoke put them at greater risk of SIDS [37]. Epidemiological research has shown that Native Americans, Maoris from New Zealand, and Aboriginal Australians have much higher rates of SIDS than non-Indigenous groups within the same nations [38].

Maternal Smoking and Smoke Exposure 

According to a recent study, the quantity of cigarettes smoked daily during pregnancy has a linear relationship with the probability of a SIDS occurrence. Also, avoiding smoking during the first three months of pregnancy is closely linked to a significant reduction in SIDS risk. Reducing the number of cigarettes smoked per day also adds to a slight reduction in risk [39, 40]. Beyond the first trimester of pregnancy, combined exposure to alcohol and smoking poses a more significant risk of the incidence of SIDS [41]. Tobacco smoke exposure during pregnancy is another major risk factor for SIDS. There is also a risk of post-natal exposure, which increases with other smokers in the family or the amount of time the infant is exposed to smoke-filled surrounding [42]. Bed-sharing adds substantially to the risk associated with maternal smoking [40]. Only a few researchers have looked into the link between substance abuse and SIDS. Substance misuse being frequently involved with multiple substances, make it hard to distinguish each effect [36].

*Sleeping Positions* 

In several Western regions, it is recommended to lie down supine and in a bed other than put nearer to adults to avoid Sudden Infant Death Syndrome (SIDS) [43]. Mother-infant bed-sharing has been known to increase the risk of infant deaths from sleep-related causes, which affect African Americans at a vastly disproportionate higher rate [44]. Rebreathing of exhaled air, with increased carbon dioxide and reduced oxygen concentration levels, has long been involved in inexplicable SIDS, which takes place when infants are positioned to nap in a prone (facedown) position. This position has also been linked with lower hypertension and impeded lower blood pressure [45,46]. Both prone and side sleeping raise the risk of SIDS. Premature babies that are put prone are just as in danger for SIDS as babies born at gestation [47]. The risk is further increased in low birth weight babies, premature babies, and infants between the ages of 13 and 24 weeks, implying that SIDS may be induced by no obvious cause. The prone position generally tends to raise stimulation and wakening thresholds, encourages sleep, as well as minimises neurogenic action via reduced parasympathetic activity, reduced sympathetic activity, or perhaps a lack of balance between two systems [47,48]. Infant baroreflex function is influenced by the infant's sleeping position, sleep stage, and post-natal age, according to various cross-spectral analyses employing flex sensitivity (BRS). Decreased BRS in early infancy who are sleeping in a prone position may make them more susceptible to hypotensive episodes when they sleep and be crucial in the circumstances like SIDS whereby circulation insufficiency may be involved [45].

Even though there is little vulnerability to environmental and genetic factors, such as cigarette smoke, soft bed sheets, prone sleep position, and co-sleeping, newborns in childcare settings, i.e., those who appear to care for by a caretaker who is not a parent, which includes caretakers and child care providers-make up about 20% of SIDS deaths. However, it is unclear why there is such a large percentage of risk infants [49-51]. Cross-sectional studies have indicated that despite the majority of healthcare professionals recognised the supine position as the position that minimises the risk of sudden infant death syndrome the most, only 30% acknowledged most frequently putting infants in that position to sleep, with the majority of staff (91%) claiming fear of aspiration as the reason for supine position avoidance [52]. Additionally, a significant number of these fatalities occur within a week of childcare [50]. Sudden infant death syndrome (SIDS) has been linked to a higher risk in infants who are overheated [53]. Thermal stress can cause mortality directly through hyperthermia (or hypothermia). It can also affect the body's central nervous system by affecting the respiratory rate, the larynx closing reflex, or the arousal processes [54,55].

Medical Conditions Affecting SIDS

Rate of SIDS incidences in various seasons: When SIDS occurrences rise in the cold season, they coincide with respiratory viral outbreaks and frequently show symptoms of respiratory system irritation and a history of mild sickness symptoms. There are physiological methods by which newborns can develop frequent or severe, potentially fatal hypoxemia. Respiratory infections are a major cause of newborns presenting with sudden events involving apnea and hypoxemia [56].

Parental room sharing: Sharing a room but not a bed is advised. It is advised to eliminate soft bedding such as pillows, blankets, and other items from the child's bedrooms [9]. Although it should be prevented in newborns in the first four weeks who are breastfeeding, pacifier use seems to reduce the incidence of sudden infant death syndrome [57]. 

Maternal smoking: Babies who are exposed to environmental tobacco smoke (ETS) are more likely to develop chronic obstructive pulmonary disease (COPD), bronchial asthma, and pneumonia, especially in the first two years of age [58]. Two to three times as many premature and low birth weight babies die abruptly and suddenly compared to normal newborns [59]. Cigarette smoke has an effect on prenatal and post-natal growth, raises the risk of infections, and increases the likelihood that perhaps the child may experience paediatric cardiac disease [60].

Genetics 

Comprehension of the genetic markers predisposing to SIDS is a significant challenge. Before a genetic cause is successfully identified, a biological disease must first be detected and thoroughly characterised in most medical disciplines. In SIDS genetic research, however, the situation has been reversed: genetic testing and study have aided in elucidating potential mechanisms of death in SIDS. This is a multi-factorial syndrome, leaving us with a rudimentary understanding of the factors which contribute to the causes leading to infant death [61]. The Triple Risk Model of SIDS suggests that genetic components play a role in SIDS, with "vulnerable infants" affected by the genetic variables [62].

Metabolic diseases: Acyl-CoA Dehydrogenase Medium Chain (ACADM) is the gene that has been studied the most concerning metabolic problems in SIDS. It catalyses the initial step in the beta-oxidation of medium-chain fatty acids [63]. Autosomal recessive mutations in this gene result in MCAD (medium-chain acyl-CoA deficiency). In another study on the genetic investigation of 161 SIDS cases in 2017, two children with gene mutations that resulted in *DOLK*-congenital disorder of glycosylation and systemic primary carnitine insufficiency were found, accounting for 1% of the overall cohort [64].

Cardiac genes: SIDS cases with monogenic genetic cardiovascular problems comprise a significant but tiny percentage of total SIDS cases [61]. A study found possibly disease-causing variations in 20% of the 155 SIDS cases by considering a list of 192 genes linked to cardiovascular and metabolic illnesses. Gene variants linked to channelopathies (9%) and cardiomyopathies (7%) were found in a majority of these patients [62]. *CAV3* (Caveolin 3), *GJA1* (Gap Junction Protein Alpha 1), *GPD1-L* (Glycerol 3-phosphate dehydrogenase 1 like gene), *KCNE2* (Potassium Voltage-Gated Channel Subfamily E Regulatory Subunit 2), *KCNJ8* (Potassium Inwardly Rectifying Channel Subfamily J Member 8), *KCNQ1* (Potassium Voltage-Gated Channel Subfamily Q Member 1), *KCNH2* (Potassium voltage-gated channel subfamily H member 2), *MYBPC3* (Myosin Binding Protein C3), *RYR2* (Ryanodine receptor 2), *SCN5A* (Sodium Voltage-Gated Channel Alpha Subunit 5), and *TNNI3* (Troponin I3, Cardiac Type) are the primary cardiac genes discovered in previous SIDS research [65]. Further studies have also described SIDS patients with a history of Brugada syndrome in the family, genetic variants linked to Brugada syndrome, and operational data supporting this [66].

The serotonin system: Researchers have looked into genes connecting the serotonin system and SIDS cases, such as FEV (the human equivalent of Pet1), TPH2 (tryptophan hydroxylase, the rate-limiting enzyme for serotonin), and HTR1A and HTR2A (serotonin receptors). Until now, no rare or damaging variants have been reported among them [67]. SIDS sufferers exhibit dysregulated autonomic function and altered neurochemistry, reduced brainstem 5-HT (5-hydroxytryptamine), tryptophan hydroxylase-2 (TPH-2), and 5-HT receptor binding and receptor expression, thereby providing proof that serotonin plays a pivotal role in SIDS [23,68-70]. 

Epilepsy: Sudden unexpected death in epilepsy (SUDEP) is the sudden, unexpected, non-traumatic death of people who have epilepsy, with or without signs of a seizure, and for whom autopsy investigation reveals no structural or toxicological cause of death [23]. *SCN1A* (Sodium voltage-gated channel alpha subunit 1), *SCN1B* (Sodium channel subunit beta-1 ), and *DEPDC5* (DEP domain-containing 5) are some of the genes for SUDEP that have been linked to SIDS [71-75]. Evidence suggests that sudden cardiac death, SIDS, and SUDEP have chromosomal complexity and a degree of commonality. In most situations, genetic risk factors and a susceptible infant are at fault [76].

Inflammation: Some cytokine gene polymorphisms may have a role in SIDS pathogenesis. However, polymorphisms are just one among the many factors resulting in SIDS risk, in addition to other intrinsic or extrinsic factors leading to SIDS [61].

**Figure 2:**
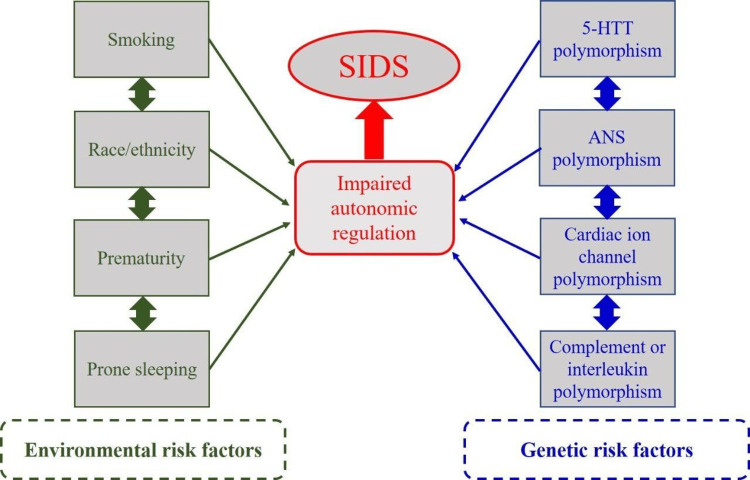
Various factors affecting SIDS SIDS: Sudden infant death syndrome; 5-HTT: 5-hydroxytryptamine (serotonin) transporter; ANS: Autonomic nervous system. Adapted from the study by Hunt CE [59].

Copy Number Variants (CNVs): One study investigating CNVs associated with SIDS discovered De novo CNVs in three of 27 SIDS patients. The significance of this discovery is unknown [77]. Figure 2 depicts an illustration of various factors affecting SIDS.

Exome or genome-wide investigations will become critical in identifying novel genotype-phenotype associations with SIDS, thereby promoting the finding of previously unknown genetic abnormalities involved in SIDS pathogenesis [61].

Diagnosis of SIDS

SIDS is defined as a diagnosis of exclusion after evaluating the medical history, complete postmortem examination, and scene investigation [78,79]. The accuracy of SIDS diagnoses is likely to be impacted by non-autopsy SIDS diagnoses. Table 1 summarizes the neurotransmitter abnormalities in the brainstem in SIDS.

**Table 1:** Neurotransmitter abnormalities in the brainstem in SIDS Adapted with permission from [96]. a.5-HT:5-hydroxytryptamine;b.ChAT:acetylcholine transferase;c.DMX: dorsal motor nucleus of the vagus; d.H.G.:hypoglossal nucleus; e.C.A.:catecholamines; f.VLM:ventrolateralmedulla;g.PNMT:phenylethanolamineN-methyltransferase; h.NTS: nucleus of the solitary tract;i.T.H: tyrosine hydroxylase;j.N.A: nucleus ambiguous; k.ICC: immunocytochemistry; l.NMDA: N-methyl-D-aspartic acid; SIDS: Sudden infant death syndrome

Substance	Effective findings
5-HT^a^	There were observed overall decreases in 5-HT1A binding, 5-HT cell proliferation, and proportionate decreases in 5-HTT [21] binding. Immunostaining of 5-HT1A and 5-HT2A receptors reveals an increase in the periaqueductal grey matter; a 5-HT rise in raphe obscures (HPLC: high-pressure liquid chromatography) was observed [80,81].
ChAT^b^	Lowering in ChAT immunostaining in DMX^c ^and HG^d^ [82].
Muscarinic receptor	A decrease in binding to the arcuate nucleus with a limited number of immunopositive neurons for muscarinic receptors in the arcuate nucleus is observed, but this has little impact on the arcuate nucleus's muscarinic receptor immunostaining [82-84].
C.A.^e^	No abnormal α2-adrenergic receptor binding was observed [85], so although dendritic spines in C.A. neurons increased in VLM^f^ [86]: PNMT^g^ immunoreactivity was absent in NTS^h^[87], T.H.^i^ immunostaining was reduced in VLM and DMX [88], and T.H. immunostaining was not correlated with the number of T.H.- immunostained neurons in DMX, NTS, NA^j^, or VLM [89].
α2-adrenergic receptor	Decreased α-2A receptors by ICC^k^ in VLM & NTS [90].
NMDA^l ^receptor	Six medulla nuclei had higher levels of NR1 subunit mRNA, higher levels of NR1 protein in DMX, and lower levels of NR1 protein in the spinal trigeminal nucleus [83].
Kainate receptor	Reduced binding in arcuate nucleus [91].
Substance P	Increased immunostaining in trigeminal fibres, higher immunostaining in NTS and spinal trigeminal nucleus, increased immunostaining in medulla homogenates, and no differences in binding in medulla [92- 95]

Histopathology

In a substantial percentage of patients, histopathological examinations have revealed subtle alterations in lung inflammation (indicating recent viral infection) [96,97]. Nevertheless, the brain occasionally exhibits minor alterations. There could be Inflammation or other changes in the intestines. Cardiomyocyte and diaphragm changes have been seen, although they do not seem to be confirmed [98,99]. Some SIDS instances have signs of a systemic inflammatory reaction.

Microbiology

In around one-fifth of instances, examination of the carcass in typically sterile places (heart blood, spleen, cerebrospinal fluid) reveals the presence of a bacterial infection (e.g., S aureus, Escherichia coli, and various coliforms) [100,101]. In SIDS cases, as opposed to non-SIDS deaths, Coliforms and S aureus were isolated from the lungs and airways more frequently [100,101].

Histology of Thymus 

It has been discovered that SIDS has a very high rate of petechial thymus haemorrhages. It was possible to demonstrate bleeding everywhere, and it was histologically restricted in a distinctive fashion almost exclusively to the cortical zones of the lobes, usually missing or just a little in non-SIDS cases [102]. 

Genetic Variations in SIDS

Maximum newborns have a variety of genetic consequences associated with channelopathies (9%), accompanied by way of cardiomyopathies (7%) and metabolic problems (1%) [61]. However, fatal arrhythmia represents a workable cause and possible cause of death. Various cardiac genes within the SIDS were observed [62].

RISK reduction interventions and their effects

Sleeping Position 

The supine position reduces the risk of SIDS. Therefore infants should be placed in the supine position while sleeping. However, there are some contraindications to placing an infant in a supine position while sleeping [103]. The prone position is recommended only when the baby is awake [104].

Sleeping in the prone position is also recommended when a baby has gastroesophageal disease, upper airway malformations, and active respiratory illness [105]. An infant placed in the prone position while sleeping has been shown to increase the risk of SIDS by 14-fold and causes additional stress on the cardiovascular and respiratory systems [106].

*Bed Sharing* 

Co-sleeping with parents, albeit still under scrutiny, has been shown in recent studies to reduce the risk of SIDS. However, co-sleeping with parents is not advisable in certain conditions that include places like sofas, and armchairs, where one/both of the parents are either smokers/alcoholics or are under medication like anxiolytic and antidepressants [35]. 

Sleep Areas

The safest place for an infant to sleep is on a separate surface designed for infants close to their parent's bed. Sleeping in the parent's room on a separate surface can reduce the risk of SIDS by 50%. It prevents suffocation, strangulation, and entrapment that may occur when the infant is sleeping in an adult bed.

Infants should be placed near the parent's bed but on a separate, detached surface for at least six months as the rate of SIDS and other sleep-related deaths are higher in the first six months of life. Infants should never be left to sleep on couches or armchairs. Bedside sleepers that are attached to parents' beds are used by some parents so that monitoring infants and breastfeeding becomes easier [107-110].

Head Coverings 

As the head is the most common site of heat production, covering an infant's head causes heat entrapment between the head surface and clothing and increases the risk of SIDS. Therefore infants' heads should be made free of coverings to reduce the risk of SIDS [111]. 

Bedding

Soft bedding plays the most common role in increasing the risk of SIDS. The surface under the infant gets depressed when the infant is placed over soft surfaces that constitute soft mattresses, sheepskin, soft pillows, and blankets and may result in suffocation, asphyxia, and overheating of the infant [112]. The risk of infant death increases if the infant is left to sleep on a couch or a sofa. A firm flat surface covered only by a thin fitted sheet should be used. Delicate items such as toys, crib bumpers, positioners, and pillows should be avoided [113].

Pacifier Use

The risk of SIDS had reduced with the usage of pacifiers. The use of pacifiers in infants older than one month is currently advised by numerous researchers to prevent sudden infant death syndrome and is related to various benefits for premature newborns, but it is also connected with a higher risk of otitis media. According to surveys, neonates have slept with pacifiers exhibited lower auditory arousal thresholds than infants who did not use pacifiers [114]. 

The pacifier should be used when placing the infants to sleep. Pacifier usage in breastfed infants isn't advisable until breastfeeding has been firmly established [115,116]. Due to the risk of strangulation, pacifiers shouldn't be placed around infants' necks. Pacifiers attached to toys or other items may lead to suffocation/choking [117].

*Breastfeeding* 

The mechanism of the beneficial effect of breastfeeding with SIDS remains unclear. In some research, breastfeeding decreases the risk of SIDS by nearly 50% (odds ratio {OR} 0.52, 95% CI: 0.46 to 0.60) after ruling out possible confounding factors [117-118]. This protection may be a consequent effect because breastfeeding has an evitable protective effect on infections, such as gastroenteritis, otitis media, and lower respiratory tract infections, which is also a risk of SIDS. Moreover, breast milk is abundant in vitamins and antibodies, which could play a role in protecting babies from vulnerable factors [117,118]. In terms of the cardiovascular system, breastfed infants have obviously lower heart rates and are more easily aroused, compared with formula-fed ones, which could also protect the babies [119,120]. Another study showed that healthy breastfed babies were more likely to awaken from active sleep than formula-fed babies in response to nasal air-jet stimulation [121].

In addition, the brains of breastfed infants have docosahexaenoic acid, a long-chain polyunsaturated fatty acid (LCPFA), which is also found in breast milk and fish oil, which may hasten changes in the neurochemical makeup of the brain. The benefits of breastfeeding are undisputed. However, the evidence of its beneficial effect in reducing the risk of SIDS is not clear. Even with the effect on the brain by LCPFA, we have already put some in formulas nowadays. However, breastfeeding is proven to be a protective factor for many reasons; it should be encouraged to keep as long as possible. Moreover, in proportion to how long the mother breastfeeds, she also benefits from its prevention of breast and ovarian malignancies, as well as type 2 diabetes [122]. 

Smoking and Alcohol Avoidance

Children born to mothers who both drank and smoked have a 12-fold increased risk for SIDS. Avoiding the use of smoke and alcohol during pre and post-natal periods decreases the risk of SIDS [35]. Table 2 depicts all of the risk reduction interventions described above, and their effects on the occurrence of SIDS.

**Table 2:** Recommendations to reduce the risk of SIDS Adapted with permission from [118]. SIDS: Sudden infant death syndrome

Risk factors	Recommendations
Bed sharing	Infants may be brought into the bed for feeding or comforting but should be returned to a separate sleep area when the parent is ready to return to sleep. It is prudent to provide separate sleep areas and avoid co-bedding for twins and other infants of multiple gestations. Room sharing without bed-sharing is recommended. Devices promoted to make bed-sharing safe are not recommended. There are specific circumstances in which bed-sharing is particularly hazardous, and it should be stressed to parents that they avoid bed-sharing during the following situations at all times: If either parent smokes If the infant is younger than three months If the infant is placed on excessively soft surfaces (e.g., waterbeds, sofas, armchairs) If soft bedding accessories (e.g., pillows, blankets) are used If there are multiple bed sharers If the parent has consumed alcohol If the infant is bed-sharing with someone who is not a parent
Bedding	Pillows, quilts, comforters, sheepskins, and other soft surfaces are hazardous when placed under the infant or when loose in the sleep environment. Wedges, positioning devices, bumper pads, and similar products are not recommended.
Breastfeeding	Breastfeeding is recommended. If a breastfeeding mother brings the infant into her bed for nursing, the infant should be returned to a separate sleep area when the mother is ready to return to sleep.
Infant monitors and apparent life-threatening events	Infant monitors should not be used to prevent SIDS. There is no evidence that apparent life-threatening events are precursors to SIDS.
Overheating and head covering	Avoid overheating and head covering in infants.
Pacifier use	Consider offering a pacifier at nap time and bedtime.
Prenatal care and postnatal exposures	Avoid alcohol and illicit drug use during pregnancy and after the infant's birth. Pregnant women should obtain regular prenatal care. Women should avoid smoking during pregnancy, and exposure to smoke in the pregnant woman's or infant's environment should be avoided.
Sleep areas	Car seats and other sitting devices are not recommended for routine sleep at home or in the hospital, particularly for young infants. Infants should sleep in a safety-approved crib, portable crib, play yard, or bassinet.
Sleep position	Sleeping in the supine position is recommended for infants to reduce the risk of SIDS; prone or side sleeping is not safe and is not advised. Once an infant can roll from the supine to the prone position and back again, he or she can remain in either position during sleep. Supervised, awake tummy time on a daily basis can promote motor development and minimize the risk of positional plagiocephaly. SIDS = sudden infant death syndrome. Information from reference 5.

## Conclusions

SIDS is a complicated, multifaceted condition, and additional study is required to fully comprehend how intrinsic susceptibility, a crucial developmental stage, and the existence of environmental risk factors interact. We can only hope that with increased knowledge and the adoption of safe sleep practices from hospitals to homes, we may reduce the incidence of SIDS, which may never be totally eradicated. Despite the fact that the origin and causation of SIDS remain unclear, parents and caregivers need to be aware that changing particular habits, practices, and interventions may affect how an event turns out in the end. It is crucial for doctors, nurses, and other healthcare providers to convey a consistent message according to updated guidelines from the American Academy of Pediatrics and address the worries and misconceptions of parents and caregivers regarding safe sleep guidelines.

## References

[1] (2022). Data and Statistics for SIDS and SUID. https://www.cdc.gov/sids/data.htm.

[2] Wennergren G, Alm B, Oyen N (1997). The decline in the incidence of SIDS in Scandinavia and its relation to risk-intervention campaigns. Acta Paediatr.

[3] (2016). Infant Deaths. Linked Birth/Infant Death Records. http://wonder.cdc.gov/lbd.html.

[4] Willinger M, James LS, Catz C (1991). Defining the sudden infant death syndrome (SIDS): deliberations of an expert panel convened by the National Institute of Child Health and Human Development. Pediatr Pathol.

[5] Shapiro-Mendoza CK, Tomashek KM, Anderson RN, Wingo J (2006). Recent national trends in sudden, unexpected infant deaths: more evidence supporting a change in classification or reporting. Am J Epidemiol.

[6] (2016). SIDS and other sleep-related infant deaths: updated 2016 recommendations for a safe infant sleeping environment. Pediatrics.

[7] Blair PS, Sidebotham P, Evason-Coombe C, Edmonds M, Heckstall-Smith EM, Fleming P (2009). Hazardous cosleeping environments and risk factors amenable to change: case-control study of SIDS in south west England. BMJ.

[8] Scragg R, Mitchell EA, Taylor BJ (1993). Bed sharing, smoking, and alcohol in the sudden infant death syndrome. New Zealand Cot Death Study Group. BMJ.

[9] Moon RY, Fu L (2012). Sudden infant death syndrome: an update. Pediatr Rev.

[10] Leach CE, Blair PS, Fleming PJ, Smith IJ, Platt MW, Berry PJ, Golding J (1999). Epidemiology of SIDS and explained sudden infant deaths. CESDI SUDI Research Group. Pediatrics.

[11] Weese-Mayer DE, Ackerman MJ, Marazita ML, Berry-Kravis EM (2007). Sudden infant death syndrome: review of implicated genetic factors. Am J Med Genet A.

[12] Steinschneider A (1972). Prolonged apnea and the sudden infant death syndrome: clinical and laboratory observations. Pediatrics.

[13] Firstman R, Talan J The Death of Innocents.

[14] Filiano JJ, Kinney HC (1994). A perspective on neuropathologic findings in victims of the sudden infant death syndrome: the triple-risk model. Biol Neonate.

[15] Plant LD, Bowers PN, Liu Q (2006). A common cardiac sodium channel variant associated with sudden infant death in African Americans, SCN5A S1103Y. J Clin Invest.

[16] Wang DW, Desai RR, Crotti L (2007). Cardiac sodium channel dysfunction in sudden infant death syndrome. Circulation.

[17] Hunt CE, Brouillette RT (1987). Sudden infant death syndrome: 1987 perspective. J Pediatr.

[18] Weese-Mayer DE, Berry-Kravis EM, Zhou L, Maher BS, Curran ME, Silvestri JM, Marazita ML (2004). Sudden infant death syndrome: case-control frequency differences at genes pertinent to early autonomic nervous system embryologic development. Pediatr Res.

[19] Bergman AB, Wiesner LA (1976). Relationship of passive cigarette-smoking to sudden infant death syndrome. Pediatrics.

[20] Funk GD, Zwicker JD, Selvaratnam R, Robinson DM (2011). Noradrenergic modulation of hypoglossal motoneuron excitability: developmental and putative state-dependent mechanisms. Arch Ital Biol.

[21] Paterson DS, Trachtenberg FL, Thompson EG (2006). Multiple serotonergic brainstem abnormalities in sudden infant death syndrome. JAMA.

[22] Kinney HC (2005). Abnormalities of the brainstem serotonergic system in the sudden infant death syndrome: A review. Pediatr Dev Pathol.

[23] Duncan JR, Paterson DS, Hoffman JM (2010). Brainstem serotonergic deficiency in sudden infant death syndrome. JAMA.

[24] Ozawa Y, Obonai T, Itoh M, Aoki Y, Funayama M, Takashima S (1999). Catecholaminergic neurons in the diencephalon and basal ganglia of SIDS. Pediatr Neurol.

[25] Hilaire G (2006). Endogenous noradrenaline affects the maturation and function of the respiratory network: possible implication for SIDS. Auton Neurosci.

[26] Fleming KA (1992). Viral respiratory infection and SIDS. J Clin Pathol.

[27] Stoltenberg L, Saugstad OD, Rognum TO (1992). Sudden infant death syndrome victims show local immunoglobulin M response in tracheal wall and immunoglobulin A response in duodenal mucosa. Pediatr Res.

[28] Stoltenberg L, Vege A, Saugstad OD, Rognum TO (1995). Changes in the concentration and distribution of immunoglobulin-producing cells in SIDS palatine tonsils. Pediatr Allergy Immunol.

[29] Thrane PS, Rognum TO, Brandtzaeg P (1994). Up-regulated epithelial expression of HLA-DR and secretory component in salivary glands: reflection of mucosal immunostimulation in sudden infant death syndrome. Pediatr Res.

[30] Highet AR, Goldwater PN (2009). Staphylococcal enterotoxin genes are common in Staphylococcus aureus intestinal flora in sudden infant death syndrome (SIDS) and live comparison infants. FEMS Immunol Med Microbiol.

[31] Rand CM, Weese-Mayer DE, Maher BS, Zhou L, Marazita ML, Berry-Kravis EM (2006). Nicotine metabolizing genes GSTT1 and CYP1A1 in sudden infant death syndrome. Am J Med Genet A.

[32] Moon RY, Horne RS, Hauck FR (2007). Sudden infant death syndrome. Lancet.

[33] Adams SM, Ward CE, Garcia KL (2015). Sudden infant death syndrome. Am Fam Physician.

[34] Stiffler D, Ayres B, Fauvergue C, Cullen D (2018). Sudden infant death and sleep practices in the Black community. J Spec Pediatr Nurs.

[35] Moon RY, Hauck FR (2018). SIDS Sudden Infant and Early Childhood Death: The Past, the Present and the Future.

[36] Maged M, Rizzolo D (2018). Preventing sudden infant death syndrome and other sleep-related infant deaths. JAAPA.

[37] Blackwell CC, Moscovis SM, Gordon AE (2004). Ethnicity, infection and sudden infant death syndrome. FEMS Immunol Med Microbiol.

[38] Anderson TM, Lavista Ferres JM, Ren SY, Moon RY, Goldstein RD, Ramirez JM, Mitchell EA (2019). Maternal smoking before and during pregnancy and the risk of sudden unexpected infant death. Pediatrics.

[39] McIntosh C, Trenholme A, Stewart J, Vogel A (2018). Evaluation of a sudden unexpected death in infancy intervention programme aimed at improving parental awareness of risk factors and protective infant care practices. J Paediatr Child Health.

[40] Elliott AJ, Kinney HC, Haynes RL (2020). Concurrent prenatal drinking and smoking increases risk for SIDS: Safe Passage Study report. EClinicalMedicine.

[41] Fleming P, Blair PS (2007). Sudden infant death syndrome and parental smoking. Early Hum Dev.

[42] Keywan C, Poduri AH, Goldstein RD, Holm IA (2021). Genetic factors underlying sudden infant death syndrome. Appl Clin Genet.

[43] Brownstein CA, Poduri A, Goldstein RD, Holm IA (2018). The Genetics of Sudden Infant Death Syndrome. SIDS Sudden Infant and Early Childhood Death: The Past, the Present and the Future.

[44] Neubauer J, Lecca MR, Russo G, Bartsch C, Medeiros-Domingo A, Berger W, Haas C (2017). Post-mortem whole-exome analysis in a large sudden infant death syndrome cohort with a focus on cardiovascular and metabolic genetic diseases. Eur J Hum Genet.

[45] Pryce JW, Weber MA, Heales S, Malone M, Sebire NJ (2011). Tandem mass spectrometry findings at autopsy for detection of metabolic disease in infant deaths: postmortem changes and confounding factors. J Clin Pathol.

[46] Courts C, Madea B (2010). Genetics of the sudden infant death syndrome. Forensic Sci Int.

[47] Cerrone M, Remme CA, Tadros R, Bezzina CR, Delmar M (2019). Beyond the one gene-one disease paradigm: complex genetics and pleiotropy in inheritable cardiac disorders. Circulation.

[48] Paterson DS (2013). Serotonin gene variants are unlikely to play a significant role in the pathogenesis of the sudden infant death syndrome. Respir Physiol Neurobiol.

[49] Haynes RL, Folkerth RD, Paterson DS (2016). Serotonin receptors in the medulla oblongata of the human fetus and infant: the analytic approach of the International Safe Passage Study. J Neuropathol Exp Neurol.

[50] Broadbelt KG, Rivera KD, Paterson DS (2012). Brainstem deficiency of the 14-3-3 regulator of serotonin synthesis: a proteomics analysis in the sudden infant death syndrome. Mol Cell Proteomics.

[51] Ravindran K, Powell KL, Todaro M, O'Brien TJ (2016). The pathophysiology of cardiac dysfunction in epilepsy. Epilepsy Res.

[52] Hu D, Barajas-Martínez H, Medeiros-Domingo A (2012). A novel rare variant in SCN1Bb linked to Brugada syndrome and SIDS by combined modulation of Na(v)1.5 and K(v)4.3 channel currents. Heart Rhythm.

[53] Le Gal F, Korff CM, Monso-Hinard C, Mund MT, Morris M, Malafosse A, Schmitt-Mechelke T (2010). A case of SUDEP in a patient with Dravet syndrome with SCN1A mutation. Epilepsia.

[54] Ramadan W, Patel N, Anazi S (2017). Confirming the recessive inheritance of SCN1B mutations in developmental epileptic encephalopathy. Clin Genet.

[55] Bagnall RD, Crompton DE, Petrovski S (2016). Exome-based analysis of cardiac arrhythmia, respiratory control, and epilepsy genes in sudden unexpected death in epilepsy. Ann Neurol.

[56] Nascimento FA, Borlot F, Cossette P, Minassian BA, Andrade DM (2015). Two definite cases of sudden unexpected death in epilepsy in a family with a DEPDC5 mutation. Neurol Genet.

[57] Goldstein RD, Kinney HC, Willinger M (2016). Sudden unexpected death in fetal life through early childhood. Pediatrics.

[58] Toruner GA, Kurvathi R, Sugalski R (2009). Copy number variations in three children with sudden infant death. Clin Genet.

[59] Hunt CE (2005). Gene-environment interactions: implications for sudden unexpected deaths in infancy. Arch Dis Child.

[60] Anuntaseree W, Mo-Suwan L, Vasiknanonte P, Kuasirikul S, Ma-A-Lee A, Choprapawon C (2008). Factors associated with bed sharing and sleep position in Thai neonates. Child Care Health Dev.

[61] Salm Ward TC, Ngui EM (2015). Factors associated with bed-sharing for African American and White mothers in Wisconsin. Matern Child Health J.

[62] Yiallourou SR, Sands SA, Walker AM, Horne RS (2011). Baroreflex sensitivity during sleep in infants: impact of sleeping position and sleep state. Sleep.

[63] Itzhak N, Greenblatt D (2019). Aerodynamic factors affecting rebreathing in infants. J Appl Physiol (1985).

[64] Galland BC, Taylor BJ, Bolton DP (2002). Prone versus supine sleep position: a review of the physiological studies in SIDS research. J Paediatr Child Health.

[65] Oyen N, Markestad T, Skaerven R (1997). Combined effects of sleeping position and prenatal risk factors in sudden infant death syndrome: the Nordic Epidemiological SIDS Study. Pediatrics.

[66] de Jonge GA, Lanting CI, Brand R, Ruys JH, Semmekrot BA, van Wouwe JP (2004). Sudden infant death syndrome in child care settings in the Netherlands. Arch Dis Child.

[67] Moon RY, Patel KM, Shaefer SJ (2000). Sudden infant death syndrome in child care settings. Pediatrics.

[68] Moon RY, Sprague BM, Patel KM (2005). Stable prevalence but changing risk factors for sudden infant death syndrome in child care settings in 2001. Pediatrics.

[69] Stastny PF, Ichinose TY, Thayer SD, Olson RJ, Keens TG (2004). Infant sleep positioning by nursery staff and mothers in newborn hospital nurseries. Nurs Res.

[70] Watson L, Potter A, Gallucci R, Lumley J (1998). Is baby too warm? The use of infant clothing, bedding and home heating in Victoria, Australia. Early Hum Dev.

[71] Fleming PJ, Azaz Y, Wigfield R (1992). Development of thermoregulation in infancy: possible implications for SIDS. J Clin Pathol.

[72] Blair PS, Platt MW, Smith IJ, Fleming PJ (2006). Sudden infant death syndrome and sleeping position in pre-term and low birth weight infants: an opportunity for targeted intervention. Arch Dis Child.

[73] Samuels M (2003). Viruses and sudden infant death. Paediatr Respir Rev.

[74] Adams SM, Good MW, Defranco GM (2009). Sudden infant death syndrome. Am Fam Physician.

[75] Dybing E, Sanner T (1999). Passive smoking, sudden infant death syndrome (SIDS) and childhood infections. Hum Exp Toxicol.

[76] Goodstein MH, Stewart DL, Keels EL, Moon RY (2021). Transition to a safe home sleep environment for the NICU patient. Pediatrics.

[77] Frank Wolf M, Bar-Zeev Y, Solt I (2018). Interventions for supporting women to stop smoking in pregnancy (Article in Hebrew). Harefuah.

[78] Shields LB, Hunsaker JC 3rd, Corey TS, Stewart D (2007). Is SIDS on the rise??. J Ky Med Assoc.

[79] Kopp N, Denoroy L, Eymin C (1994). Studies of neuroregulators in the brain stem of SIDS. Biol Neonate.

[80] Ozawa Y, Okado N (2002). Alteration of serotonergic receptors in the brain stems of human patients with respiratory disorders. Neuropediatrics.

[81] Mallard C, Tolcos M, Leditschke J, Campbell P, Rees S (1999). Reduction in choline acetyltransferase immunoreactivity but not muscarinic-m2 receptor immunoreactivity in the brainstem of SIDS infants. J Neuropathol Exp Neurol.

[82] Panigrahy A, Filiano JJ, Sleeper LA (1997). Decreased kainate receptor binding in the arcuate nucleus of the sudden infant death syndrome. J Neuropathol Exp Neurol.

[83] Nachmanoff DB, Panigrahy A, Filiano JJ (1998). Brainstem 3H-nicotine receptor binding in the sudden infant death syndrome. J Neuropathol Exp Neurol.

[84] Kopp N, Chigr F, Denoroy L, Gilly R, Jordan D (1993). Absence of adrenergic neurons in nucleus tractus solitarius in sudden infant death syndrome. Neuropediatrics.

[85] Obonai T, Yasuhara M, Nakamura T, Takashima S (1998). Catecholamine neurons alteration in the brainstem of sudden infant death syndrome victims. Pediatrics.

[86] Sawaguchi T, Patricia F, Kadhim H, Groswasser J, Sottiaux M, Nishida H, Kahn A (2003). The correlation between serotonergic neurons in the brainstem and sleep apnea in SIDS victims. Early Hum Dev.

[87] Sawaguchi T, Ozawa Y, Patricia F (2003). Catecholaminergic neurons in the brain-stem and sleep apnea in SIDS victims. Early Hum Dev.

[88] Machaalani R, Waters KA (2003). NMDA receptor 1 expression in the brainstem of human infants and its relevance to the sudden infant death syndrome (SIDS). J Neuropathol Exp Neurol.

[89] Kinney HC, Filiano JJ, Assmann SF (1998). Tritiated-naloxone binding to brainstem opioid receptors in the sudden infant death syndrome. J Auton Nerv Syst.

[90] Bergström L, Lagercrantz H, Terenius L (1984). Post-mortem analyses of neuropeptides in brains from sudden infant death victims. Brain Res.

[91] Jordan D, Kermadi I, Rambaud C, Bouvier R, Dijoud F, Martin D, Kopp N (1997). Autoradiographic distribution of brainstem substance P binding sites in humans: ontogenic study and relation to sudden infant death syndrome (SIDS). J Neural Transm (Vienna).

[92] Rambaud C, Guibert M, Briand E, Grangeot-Keros L, Coulomb-L'Herminé A, Dehan M (1999). Microbiology in sudden infant death syndrome (SIDS) and other childhood deaths. FEMS Immunol Med Microbiol.

[93] Weber MA, Hartley JC, Ashworth MT, Malone M, Sebire NJ (2010). Virological investigations in sudden unexpected deaths in infancy (SUDI). Forensic Sci Med Pathol.

[94] Kinney HC, Richerson GB, Dymecki SM, Darnall RA, Nattie EE (2009). The brainstem and serotonin in the sudden infant death syndrome. Annu Rev Pathol.

[95] Variend S, Sunderland R (1984). Small intestinal mucosal abnormalities in post-perinatal deaths. J Clin Pathol.

[96] Kariks J (1988). Cardiac lesions in sudden infant death syndrome. Forensic Sci Int.

[97] Kariks J. (1989 Diaphragmatic muscle fibre necrosis in SIDS. Forensic science international.

[98] Weber MA, Klein NJ, Hartley JC, Lock PE, Malone ME, Sebire NJ (2008). Infection and sudden unexpected death in infancy: a systematic retrospective case review. Lancet.

[99] Goldwater PN (2009). Sterile site infection at autopsy in sudden unexpected deaths in infancy. Arch Dis Child.

[100] Risse M, Weiler G (1989). Comparative histologic studies of the origin of petechial thymus hemorrhage (Article in German). Z Rechtsmed.

[101] Tablizo MA, Jacinto P, Parsley D, Chen ML, Ramanathan R, Keens TG (2007). Supine sleeping position does not cause clinical aspiration in neonates in hospital newborn nurseries. Arch Pediatr Adolesc Med.

[102] Laughlin J, Luerssen TG, Dias MS (2011). Prevention and management of positional skull deformities in infants. Pediatrics.

[103] Sánchez Ruiz-Cabello J (2023). Prevention of sudden infant death syndrome. https://previnfad.aepap.org/monografia/muerte-subita-lactante.

[104] Mitchell EA, Freemantle J, Young J, Byard RW (2012). Scientific consensus forum to review the evidence underpinning the recommendations of the Australian SIDS and Kids Safe Sleeping Health Promotion Programme--October 2010. J Paediatr Child Health.

[105] Carpenter RG, Irgens LM, Blair PS (2004). Sudden unexplained infant death in 20 regions in Europe: case control study. Lancet.

[106] Blair PS, Fleming PJ, Smith IJ (1999). Babies sleeping with parents: case-control study of factors influencing the risk of the sudden infant death syndrome. CESDI SUDI research group. BMJ.

[107] Tappin D, Ecob R, Brooke H (2005). Bedsharing, roomsharing, and sudden infant death syndrome in Scotland: a case-control study. J Pediatr.

[108] Jardine DS (1992). A mathematical model of life-threatening hyperthermia during infancy. J Appl Physiol (1985).

[109] Carlin RF, Moon RY (2017). Risk factors, protective factors, and current recommendations to reduce sudden infant death syndrome: a review. JAMA Pediatr.

[110] Hauck FR, Omojokun OO, Siadaty MS (2005). Do pacifiers reduce the risk of sudden infant death syndrome? A meta-analysis. Pediatrics.

[111] Franco P, Scaillet S, Wermenbol V, Valente F, Groswasser J, Kahn A (2000). The influence of a pacifier on infants' arousals from sleep. J Pediatr.

[112] Li DK, Willinger M, Petitti DB, Odouli R, Liu L, Hoffman HJ (2006). Use of a dummy (pacifier) during sleep and risk of sudden infant death syndrome (SIDS): population based case-control study. BMJ.

[113] (2012). Breastfeeding and the use of human milk. Pediatrics.

[114] Hoffman HJ, Damus K, Hillman L, Krongrad E (1988). Risk factors for SIDS. Results of the National Institute of Child Health and Human Development SIDS cooperative epidemiological study. Ann N Y Acad Sci.

[115] Vennemann MM, Bajanowski T, Brinkmann B, Jorch G, Yücesan K, Sauerland C, Mitchell EA (2009). Does breastfeeding reduce the risk of sudden infant death syndrome?. Pediatrics.

[116] Hauck FR, Thompson JM, Tanabe KO, Moon RY, Vennemann MM (2011). Breastfeeding and reduced risk of sudden infant death syndrome: a meta-analysis. Pediatrics.

[117] Gordon AE, Saadi AT, MacKenzie DA (1999). The protective effect of breast feeding in relation to sudden infant death syndrome (SIDS): III. Detection of IgA antibodies in human milk that bind to bacterial toxins implicated in SIDS. FEMS Immunol Med Microbiol.

[118] McVea KL, Turner PD, Peppler DK (2000). The role of breastfeeding in sudden infant death syndrome. J Hum Lact.

[119] Butte NF, Smith EO, Garza C (1991). Heart rate of breast-fed and formula-fed infants. J Pediatr Gastroenterol Nutr.

[120] Elias MF, Nicolson NA, Bora C, Johnston J (1986). Sleep/wake patterns of breast-fed infants in the first 2 years of life. Pediatrics.

[121] Horne RS, Franco P, Adamson TM, Groswasser J, Kahn A (2004). Influences of maternal cigarette smoking on infant arousability. Early Hum Dev.

[122] Aguiar H, Silva AI (2011). Breastfeeding: the importance of intervening (Article in Portuguese). Acta Med Port.

